# Development of an HFACS-based risk assessment indicator system for unplanned endotracheal extubation in adult ICU patients: a Delphi and AHP study

**DOI:** 10.3389/fmed.2026.1922086

**Published:** 2026-10-28

**Authors:** Cuixue Wang, Wei Cao, Yuan Yuan, Jing Li, Yana Xing, Xiaozhe Hu, Ran Zhang

**Affiliations:** 1 Department of Critical Care Medicine, Beijing Tiantan Hospital of Capital Medical University, Beijing, China; 2 Nursing Department, Beijing Tiantan Hospital of Capital Medical University, Beijing, China

**Keywords:** analytic hierarchy process, Delphi method, human factors analysis and classification system (HFACS), patient safety risk, unplanned endotracheal extubation (UEE)

## Abstract

**Objective:**

To construct a risk-assessment indicator system for unplanned endotracheal extubation (UEE) among adult ICU patients based on the Human Factors Analysis and Classification System (HFACS), and provide a structured multi-level framework for unit-level safety auditing and root-cause analysis of UEE events.

**Methods:**

Initial indicators were generated via systematic literature review and retrospective analysis of local UEE adverse-event cases. A two-round Delphi expert consultation was performed to screen and revise indicators. The Analytic Hierarchy Process (AHP) was adopted to calculate indicator weights at each hierarchical level. Content validity was evaluated using the content validity index (CVI).

**Results:**

The valid response rates for the two Delphi rounds were 100.0% (20/20) and 95.0% (19/20), respectively. The expert authority coefficients (Cr) were 0.893 and 0.915, indicating high expert authority. Kendall's coefficients of concordance (*W*) were 0.242 and 0.254 (*P* < 0.001), showing statistically significant yet low expert agreement. Among the four primary dimensions, the combined weights were 0.448 for unsafe behavior of clinicians, 0.257 for inadequate supervision, 0.151 for preconditions for unsafe acts, and 0.144 for organizational influence. The final risk-assessment indicator system comprised 4 primary, 13 secondary, and 63 tertiary indicators with acceptable overall content validity.

**Conclusion:**

Using HFACS, two-round Delphi consultation, and AHP, this study developed a consensus-driven preliminary indicator system for UEE risk-related safety management in adult ICUs. This framework is intended for system-oriented safety audits, training priority setting, and root-cause analysis rather than bedside individual-patient risk prediction. Further prospective clinical validation is required to verify its predictive validity, reliability and practical feasibility before widespread clinical implementation.

## Practitioner points

- Systematic framework to integrate behavioral, environmental, supervisory, and organizational layers for UEE risk identification using the HFACS model.
- Delphi-AHP process quantified expert ratings on the importance of 63 risk indicators
- The indicator system can guide resource allocation, training prioritization, and safety protocol development, but requires prospective validation before use as a bedside risk prediction tool.

## Management relevance for clinical practice

- Built upon the HFACS framework, this consensus-driven indicator system offers ICU managers a multi-layered safety-audit tool for unplanned endotracheal extubation, rather than a validated individual-patient risk-prediction instrument.
- Practically, the framework can support post-incident root-cause analysis to uncover latent system-level vulnerabilities instead of focusing solely on individual clinician errors. It also facilitates evidence-informed priority-setting for quality-improvement activities and staff training guided by AHP-derived indicator weights.
- Adaptive local adjustments and prospective validation are required before widespread clinical implementation.

## 1 Introduction

Tracheal intubation is a crucial support technique for resuscitation, treatment and life maintenance in critically ill patients in the intensive care unit (ICU). However, the varying levels of discomfort experienced by patients during intubation and inappropriate medical or nursing procedures increase the likelihood of unplanned endotracheal extubation (UEE) (1). UEE occurs when an endotracheal tube is inadvertently removed from the trachea without intentional action or directive from a healthcare provider (2). A systematic review and meta-analysis reported a pooled prevalence of UEE of 6.69% (95% CI: 5.29%–8.24%) among adult ICU patients, with an incidence density of 1.06 events per 100 ventilator-days (3). In China, data from 9,330 UEE cases reported by tertiary hospitals between 2019 and 2022 showed that patient self-extubation accounted for 74.6% of cases (4). The reported incidence of UEE in Chinese ICUs varies widely across studies, ranging from 4.5% to 22.1% (5).

UEE may result in severe complications such as asphyxiation, arrhythmias, bronchospasm, and aspiration pneumonia. Although the hospital mortality rate among patients who experience UEE has been reported to range from 10% to 25% (6), recent evidence suggests that UEE itself may not be independently associated with increased mortality. Rather, mortality risk is primarily driven by the need for reintubation and the severity of underlying illness (7). Developing a structured risk identification framework may assist clinicians in systematically recognizing risk factors and implementing targeted preventive strategies, with the goal of reducing UEE incidence.

While numerous studies and practical efforts globally have focused on identifying and managing the risk factors influencing UEE occurrence, few have systematically synthesized and comprehensively evaluated and integrated these factors using a theoretical framework. The Human Factors Analysis and Classification System (HFACS) categorizes the causes of unsafe events from overt behaviors to underlying causes into four levels: unsafe acts of operators (Level 1), preconditions for unsafe acts (Level 2), unsafe supervision (Level 3), and organizational influences (Level 4). Each lower level is influenced by the levels above, allowing for a more specific analysis and clearer categorization of causes (8), thus offering innovative approaches and methods to mitigate the impact of human factors on medical safety. For instance, the HFACS framework has been successfully adapted to healthcare settings to analyze adverse events such as surgical errors and medication incidents (9). Gao et al. (10) developed an HFACS-based risk assessment system for needlestick injuries, demonstrating robust clinical applicability. However, systematic HFACS-based risk-identification frameworks for UEE in ICU patients remain notably underexplored.

This study was conducted in an ICU with 92 beds that primarily admits neurosurgical and neurological patients. The nurse-to-patient ratio was 2.36:1, and standardized sedation and restraint management protocols were implemented. The baseline incidence of unplanned extubation in the hospital was 2.89%.

This study, grounded in the HFACS framework, integrated findings from a literature review and a retrospective case analysis to draft an initial indicator pool. Through two rounds of Delphi expert consultations and the Analytic Hierarchy Process (AHP), we developed a consensus-based risk assessment indicator system for UEE specifically in adult patients who are mechanically ventilated via an endotracheal tube in the ICU, comprising 4 primary, 13 secondary, and 63 tertiary indicators. The system incorporates four hierarchical dimensions,thereby extending beyond conventional single-level analyses to provide a multi-dimensional framework for organizing UEE-related risk factors. The AHP was further applied to assign weights to each indicator based on expert pairwise comparisons. It should be noted that this is a framework rather than a validated clinical prediction tool; the findings provide initial support for its content validity, while its predictive validity and clinical utility require future empirical testing.

## 2 Methods

### 2.1 Establishment of the research team

The research team comprised eight members with expertise in nursing management, critical care nursing, and nursing research. The team included one chief nurse, who provided overall project supervision; two associate chief nurses, who were responsible for study design, expert screening and liaison, and quality assurance; two senior nurses, who drafted the initial indicator system, distributed and collected questionnaires, and organized the data; one nurse, who collected and synthesized expert opinions; two nursing graduate students, who performed literature searches, reviewed relevant studies, and conducted statistical analyses. This study was approved by the Ethics Committee of Beijing Tiantan Hospital, Capital Medical University (approval number: KY2023-236-02).

### 2.2 Drafting the initial UEE indicator system

#### 2.2.1 Literature review and indicator extraction

A literature search was conducted to identify risk factors for UEE in adult ICU patients. The search was performed in Chinese databases (CNKI, Wanfang, VIP) and English databases (PubMed, EMBASE, Web of Science, CINAHL) from database inception to January 1, 2023. Search terms combined subject headings and free words across three domains: (1) population: “intensive care”/“critically ill”/“ICU”; (2) exposure: “tracheal intubation”/“mechanical ventilation”; and (3) outcome: “unplanned extubation”/“UEE”/“self-extubation”/“accidental extubation”/“dislodgement”. These were combined with Boolean operators AND/OR. The complete search strategies for each database are provided in Supplementary Table S1.

Two research team members independently screened titles and abstracts, followed by full-text review; disagreements were resolved through discussion or adjudication by a third senior reviewer. Studies were included if they: (1) involved adult ICU patients (≥18 years) receiving endotracheal intubation; (2) reported risk factors, management strategies, or assessment tools related to UEE; (3) were published in peer-reviewed journals; and (4) were available in English or Chinese. Exclusion criteria were: (1) studies focused exclusively on pediatric or neonatal populations; (2) case reports, editorials, or conference abstracts; (3) studies on tracheostomy or non-invasive ventilation; and (4) duplicate publications.

A total of 2,548 records were identified. After removing duplicates and applying the eligibility criteria, 19 articles were included after evaluating their quality. These comprised four guidelines (11–14), one expert consensus (15), one best practice (16), one summary of evidence (17), nine systematic reviews (18–26), two scale development (1, 27), one randomized controlled study (28). The screening process and reasons for exclusion at each stage are detailed in Supplementary Table S2.

Two researchers independently assessed the methodological quality of the included studies, with disagreements resolved through discussion or adjudication by a third reviewer. The appraisal tools were selected according to study type: guidelines and consensus statements were evaluated using the AGREE II instrument; systematic reviews and meta-analyses were assessed using AMSTAR 2; best practice recommendations and evidence summaries were appraised using the JBI Checklist for Text and Opinion Papers; and scale development studies were evaluated using the JBI Checklist for Analytical Cross-Sectional Studies. For AGREE II, a standardised domain score ≥60% in all domains was considered high quality. For AMSTAR 2, satisfaction of all 7 critical items was rated as high quality, 5–6 critical items as moderate, and ≤4 as low quality. For JBI checklists, a total score ≥75% was considered high quality.

Based on the original classifications in these papers and further analysis, relevant indicators were extracted and grouped.

#### 2.2.2 Retrospective case review

To supplement the literature review findings with institution-specific data, the research team accessed the hospital's adverse event reporting system and conducted a retrospective review of all documented UEE cases occurring in the study hospital's ICUs over a five-year period (January 2018 to December 2022). Cases were included if they involved adult patients (≥18 years) who had experienced an unplanned endotracheal extubation during their ICU stay. UEE was defined as any unintended removal of an endotracheal tube from the trachea, consistent with the definition used in the hospital's incident reporting system. During this period, a total of 17 UEE events were identified among adult patients receiving endotracheal intubation, yielding a UEE incidence rate of 2.89% (calculated as the number of UEE events divided by the total number of intubated patients during the same period, multiplied by 100). The denominator data were extracted from the hospital's electronic health record system.

For each identified case, two team members independently extracted data on patient demographics, clinical characteristics, event circumstances (including time of day and patient activity), and contributing factors as documented in incident reports; disagreements were resolved through discussion with a third reviewer. The extracted factors were then integrated with the literature-derived indicators and mapped onto the HFACS framework's four hierarchical levels.

Following team discussions to consolidate overlapping items, the initial indicator pool was drafted, comprising 4 primary, 13 secondary, and 82 tertiary indicators.

### 2.3 Implementation of the Delphi expert consultation

#### 2.3.1 Questionnaire development

The expert consultation questionnaire was structured into four sections. The first section provided an introduction to the study background, objectives, and instructions for completing the questionnaire. The second section presented the preliminary draft of the UEE risk assessment indicator system, wherein each indicator was rated for importance on a 5-point Likert scale ranging from 1 (unimportant) to 5 (very important). Space was also provided for experts to suggest modifications or propose additional indicators. The third section comprised an expert authority self-assessment form, which evaluated the experts' familiarity with the indicators and the basis for their judgments. The fourth section collected demographic and professional background information of the participating experts.

Following the two Delphi rounds, experts completed a separate AHP pairwise comparison questionnaire based on the finalized hierarchical structure, using Saaty's 1–9 scale. The same 19 experts who completed the second-round Delphi survey participated in this AHP pairwise comparison assessment.

#### 2.3.2 Expert selection

Twenty experts in the fields of critical care nursing, nursing management, and clinical nursing were purposively recruited from 10 provinces across China to participate in the Delphi consultation. Among them, 9 (45.0%) were from the same hospital as the research team, while the remaining 11 (55%) were from other institutions; none were members of the research team. The inclusion criteria for experts were: (1) a bachelor's degree or higher; (2) more than 10 years of clinical nursing or nursing management experience; (3) an intermediate or higher professional title; (4) willingness to participate voluntarily and to provide objective and informed responses.

The sample size of 20 experts was based on Delphi methodological guidelines recommending 10–20 experts for homogeneous panels (29). All experts provided informed consent by completing and returning the questionnaire. To ensure the independence and authenticity of responses, the consultation was conducted anonymously; experts were unaware of each other's identities, and the research team strictly protected all personal information. In the second round, one expert (5.0%) did not respond due to a business trip; the remaining 19 experts completed both rounds.

#### 2.3.3 Delphi consultation process

Two rounds of expert consultation were conducted between August and October 2023.

Prior to the first round, an *a priori* consensus threshold was established: indicators were retained if they simultaneously met three criteria: (1) importance score mean ≥4.0; (2) full score rate >60%; and (3) coefficient of variation (CV) ≤0.25 (10). Indicators not meeting these criteria were discussed by the research team with experts' comments to decide on deletion, modification, or addition; all revised indicators proceeded to the second round.

In the first round, questionnaires were distributed to the 20 selected experts via email or WeChat, with a 2-week deadline. The team analyzed responses, calculated mean scores, full score rates, and CVs for each indicator, synthesized written comments, and refined the system accordingly.

One week after Round 1, Round 2 questionnaires were distributed. Each expert received standardized feedback containing first-round statistical results, their own prior ratings, and a summary of team revisions with explanations. Experts re-rated each indicator and provided additional comments.

Final indicator weights were subsequently calculated using the Analytic Hierarchy Process (AHP) based on the second-round ratings.

### 2.4 Statistical methods

Statistical analyses were performed using Excel 2019, SPSS 24.0, and Yaahp 10.3 software.

#### 2.4.1 Descriptive statistics

Expert demographic characteristics were summarized using frequencies and proportions. Expert engagement was assessed by the questionnaire response rate and the proportion of experts providing constructive suggestions (30).

#### 2.4.2 Delphi consensus statistics

The expert positive coefficient was represented by the effective questionnaire response rate. Expert authority was measured using the authority coefficient (Cr), calculated as the arithmetic mean of the judgment coefficient (Ca) and the familiarity coefficient (Cs), with Cr ≥0.70 considered acceptable (31). The concentration of expert opinions was expressed by the full-score rate and the mean importance score. The degree of consensus among experts was assessed using the coefficient of variation (CV) and Kendall's coefficient of concordance (*W*). CV reflects the dispersion of experts' importance ratings for each indicator; a smaller CV indicates greater consistency among expert opinions. A *P*-value <0.05 for the significance test of *W* was considered statistically significant.

Content validity was assessed using a 5-point Likert scale (1 = not relevant, 2 = somewhat relevant, 3 = neutral/partially relevant, 4 = relevant, 5 = highly relevant). The item-level content validity index (I-CVI) was calculated as the proportion of experts rating an item as 4 or 5. The scale-level content validity index/average (S-CVI/Ave) was calculated as the mean of all I-CVI values. An I-CVI ≥ 0.78 and S-CVI/Ave ≥ 0.90 were considered acceptable.

#### 2.4.3 AHP weight calculation

The AHP was employed to determine indicator weights at each level. A hierarchical structure model was constructed based on the finalized indicator system. 19 Delphi experts completed pairwise comparison matrices using the Saaty 1–9 scale; individual judgments were aggregated by the geometric mean method. Local weights were obtained from normalized eigenvectors, and global weights were calculated by multiplying local weights layer by layer. Consistency was tested using the consistency ratio (CR = CI/RI), with CR <0.1 considered acceptable. Because CR reflects the overall logical consistency of a judgment matrix at a given level rather than a property of any single indicator, the range (or maximum) of CR values for each level was reported in the results instead of listing a CR for each individual indicator. Final indicator weights were then derived from the aggregated judgment matrices.

## 3 Results

### 3.1 Quality appraisal results of the included literature

Among the 19 included studies, 5 were guidelines or consensus statements: 3 international guidelines were rated as high quality by AGREE II, while 2 domestic guidelines/consensus statements were rated as moderate quality. Of the 8 systematic reviews/meta-analyses, 3 were rated as high quality, 3 as moderate quality, and 2 as low quality, primarily due to early publication and incomplete methodological reporting. The 2 best practice/evidence summary studies and the 2 scale development studies were all rated as high quality by the JBI checklists. One scoping review was included as background reference without formal quality appraisal, and 1 RCT was rated as high quality. Overall, the included studies were predominantly of moderate to high quality, providing a reliable evidence base for the development of the indicator system.

### 3.2 Demographic characteristics of the expert panel

A total of 20 experts from 10 regions across China—including Beijing, Hunan, Hubei, Fujian, Jiangsu, Shandong, Henan, Anhui, Shaanxi, and Xinjiang—participated in this Delphi consultation. The demographic and professional characteristics of the expert panel are summarized in Table 1.

**Table 1:** Demographic and professional characteristics of the expert panel (*n* = 20).

Characteristic	Category	*n*	%
Gender	Male	2	10.0
	Female	18	90.0
Age	<40 years	7	35.0
	40–50 years	10	50.0
	≥50 years	3	15.0
Education	Doctorate	1	5.0
	Master's	8	40.0
	Bachelor's	11	55.0
Professional title	Senior	3	15.0
	Associate Senior	15	75.0
	Intermediate	2	10.0
Years of clinical experience	10–20 years	10	50.0
	20–30 years	4	20.0
	≥30 years	6	30.0
Primary research area	Nursing Management	8	40.0
	Critical care nursing	9	45.0
	Clinical nursing	3	15.0

### 3.3 Expert participation

In the first consultation round, 20 questionnaires were distributed and all 20 returned, yielding a 100% response and a 100% effective response rate. Fourteen experts (70.0%) provided written suggestions for indicator revision. In the second round, 19 of 20 questionnaires were returned, representing a 95.0% response rate and a 100% effective response rate. Six experts (31.6%) offered further constructive feedback. Response rates exceeding 70% in both rounds indicate a high level of expert engagement (10).

### 3.4 Expert authority

For the two rounds, the Ca values were 0.975 and 0.980, while the Cs were 0.810 and 0.850, respectively. The resulting Cr were 0.893 and 0.915, both exceeding the recommended threshold of 0.70, indicating a high level of expert authority and supporting the reliability of the consultation findings.

### 3.5 Consensus among experts

In the first round, CV values ranged from 0 to 0.280, and Kendall's *W* was 0.242 (*P* < 0.001). In the second round, CV values ranged from 0 to 0.173, and Kendall's *W* was 0.254 (*P* < 0.001). While both W values were statistically significant, they indicated only a low level of consensus among the experts. The reduction in the maximum CV (from 0.280 to 0.173) suggests improved consistency of expert ratings following the first-round revisions. Detailed consensus statistics are presented in Table 2.

**Table 2:** Consensus statistics for expert opinions across two Delphi rounds.

Round	Indicator level	Number of indicators	Kendall's *W*	Chi-square value	*P*-value
First round (*n* = 20)	Level 1	4	0.389	29.564	<0.001
	Level 2	13	0.246	60.762	<0.001
	Level 3	71	0.234	315.666	<0.001
	Overall	88	0.242	404.624	<0.001
Second round (*n* = 19)	Level 1	4	0.295	16.811	<0.001
	Level 2	13	0.450	102.605	<0.001
	Level 3	63	0.296	348.715	<0.001
	Overall	80	0.254	400.658	<0.001

### 3.6 Establishment of the risk assessment indicator system

Following two rounds of Delphi consultation, the indicator system was refined based on statistical criteria, expert written comments, literature review findings, and team discussions. The modifications made after each round are detailed below.

#### 3.6.1 Round 1 modifications

One first-level indicator was revised from “Nurse Unsafe Behavior” to “Clinician Unsafe Behavior” to encompass both nurses and physicians. One second-level indicator was adjusted from “Nurse Condition” to “Clinician Condition.” Eleven tertiary-level indicators were updated, improve clarity and clinical relevance. For example, “Nurse has less than 3 years of work experience” was replaced with “Clinicians lack clinical experience in managing patients with tracheal intubation.”

Similarly, “Nurse has too many nursing tasks at the same time” was refined to “Clinicians have excessive concurrent tasks,” and “Nurse's assessment and decision-making on the condition of patients with tracheal intubation is insufficient” was revised to “clinicians’ assessment and decision-making regarding tracheal intubation depth, cuff pressure, and fixation effectiveness is insufficient.”

Deleted indicators: Fifteen third-level indicators were removed for failing to meet the predefined retention criteria (selection rate < 80%). These included “Nurse holds a bachelor's degree or lower,” “Economic issues,” and “Patient undergoing early mobilization.”

Added indicators: Four new tertiary-level indicators were incorporated based on expert suggestions, including “Physician's inappropriate selection of tracheal intubation tube size,” “Physician's inappropriate choice of sedation and analgesia strategy,” “Clinicians’ improper judgment of extubation criteria,” and “Patient with excessive facial sweating and oiliness.”

Merged indicators: Two third-level indicators were combined. For example, “Replacing bite block and fixation tape alone” and “Performing oral care for patients with tracheal intubation alone” were merged into “Nurse performing procedures alone, such as replacing bite block and fixation tape or oral care.”

These modifications enhanced the comprehensiveness and clinical relevance of the indicator system, ensuring broader coverage of UEE-related risk factors in ICU settings.

#### 3.6.2 Round 2 modifications

Revised indicators: Three tertiary indicators were adjusted: “Clinicians experience alarm fatigue” was updated to “Clinicians fail to promptly identify and address equipment alarms” to better reflect actionable clinical behaviors; “Family issues” was refined to “Family-related distress”; and “Emotional exhaustion” was changed to “Professional burnout” for terminological consistency with the literature.

Newly added indicators: Based on expert feedback, three new tertiary indicators were introduced: “Clinicians fail to promptly identify patient needs,” “Clinicians lack awareness of preventing accidental extubation,” and “Clinicians’ assessment and decision-making regarding patient agitation and irritability are insufficient.”

Merged indicators: Two training-related tertiary indicators were merged, and personal factor-related indicators were also combined to reduce redundancy and address ethical sensitivities regarding the recording of personal health information.

Deleted indicators: Six items were removed.

Following Round 2, expert opinions demonstrated strong alignment, and no further modifications were deemed necessary. The final risk assessment indicator system comprises 4 primary indicators, 13 secondary indicators, and 63 tertiary indicators (Table 3).

**Table 3:** Final risk assessment indicator system for UEE in ICU patients (*n* = 19).

Indicator	Importance score (mean ± SD)	CV	Full score rate (%)	Weight	Combined weight	CR
1. Unsafe Acts	5.00	0	100.0	0.448	0.448	0.025
1.1 Skill Errors	4.95 ± 0.23	0.046	94.74	0.255	0.115	0.011
1.1.1 Clinicians lack the necessary clinical experience in managing patients with tracheal intubation.	4.95 ± 0.23	0.046	94.74	0.173	0.0200	0.014
1.1.2 Clinicians are unable to promptly identify and address equipment alarms.	4.95 ± 0.23	0.046	94.74	0.247	0.028	0.014
1.1.3 Clinicians lack awareness regarding UEE prevention.	5	0	100%	0.147	0.017	0.014
1.1.4 Clinicians fail to provide adequate health education to patients.	4.79 ± 0.42	0.087	78.95	0.104	0.012	0.014
1.1.5 Clinicians are slow in identifying patient needs.	4.68 ± 0.48	0.102	68.42	0.237	0.027	0.014
1.1.6 Clinicians demonstrate incomplete mastery of standard operating procedures for tracheal intubation care.	4.95 ± 0.23	0.046	94.74	0.093	0.011	0.014
1.2 Decision-Making Errors	4.95 ± 0.23	0.046	94.74	0.243	0.109	0.011
1.2.1 Clinicians make inappropriate decisions regarding tracheal tube size selection.	4.68 ± 0.48	0.102	68.42	0.062	0.007	0.020
1.2.2 Clinicians select inappropriate sedation and analgesia methods for patients.	4.89 ± 0.32	0.064	89.47	0.108	0.012	0.020
1.2.3 Clinicians lack sufficient assessment and decision-making skills in relation to patient agitation and irritability.	4.95 ± 0.23	0.102	94.74	0.223	0.024	0.020
1.2.4 Clinicians make incorrect judgments concerning extubation criteria.	4.89 ± 0.32	0.064	89.47	0.127	0.014	0.020
1.2.5 Clinicians lack proper assessment and decision-making skills regarding protective restraint use.	4.95 ± 0.23	0.046	94.74	0.197	0.022	0.020
1.2.6 Clinicians exhibit inadequate assessment and decision-making regarding the environment.	4.53 ± 0.61	0.135	57.89	0.122	0.013	0.020
1.2.7 Clinicians lack proper assessment and decision-making during high-risk periods for UEE (e.g., nighttime, 1 h before and after shift changes, before planned extubation, during patient turning).	4.89 ± 0.32	0.064	89.47	0.160	0.017	0.020
1.3 Violations	5.00	0	100.00	0.502	0.225	0.011
1.3.1 Clinicians fail to accurately assess and communicate airway status during handover.	4.79 ± 0.42	0.087	78.95	0.200	0.045	0.014
1.3.2 Clinicians fail to restrain patients who need restraint effectively.	5.00	0	100.00	0.339	0.076	0.014
1.3.3 Clinicians fail to promptly assess tracheal intubation depth, cuff pressure, and fixation effectiveness.	5.00	0	100.00	0.331	0.075	0.014
1.3.4 Clinicians perform operations alone, such as replacing bite blocks, fixation tape, or oral care.	4.95 ± 0.23	0.046	94.74	0.130	0.029	0.014
2. Preconditions for Unsafe Acts	4.79 ± 0.41	0.096	73.68	0.151	0.151	0.025
2.1 Clinicians Condition	4.79 ± 0.42	0.087	78.95	0.396	0.060	0.015
2.1.1 Clinician fatigue and physical discomfort	4.84 ± 0.37	0.077	84.21	0.334	0.020	0.010
2.1.2 Uncorrected or inadequately corrected vision (e.g., not wearing prescribed glasses).	4.68 ± 0.48	0.102	68.42	0.192	0.012	0.010
2.1.3 Mental tension/anxiety.	4.47 ± 0.61	0.137	52.63	0.239	0.014	0.010
2.1.2 Uncorrected or inadequately corrected vision (e.g., not wearing prescribed glasses).	4.68 ± 0.48	0.102	68.42	0.1918	0.0115	0.0102
2.1.3 Mental tension/anxiety.	4.79 ± 0.54	0.112	84.21	0.2351	0.0140	0.0102
2.1.4 Professional burnout.	4.68 ± 0.48	0.102	68.42	0.192	0.012	0.010
2.2 Patient Condition	4.74 ± 0.45	0.096	73.68	0.192	0.029	0.015
2.2 Patient Condition	4.58 ± 0.77	0.168	73.68	0.0106	0.0003	0.0900
2.2.1 Age >65 years (32)	4.32 ± 0.82	0.190	52.63	0.008	0.000	0.090
2.2.2 Male gender.	4.58 ± 0.77	0.168	73.68	0.011	0.000	0.090
2.2.3 APACHE II ≥17 (33)	4.74 ± 0.45	0.096	73.68	0.062	0.002	0.090
2.2.4 Presence of comorbidities, such as congestive heart failure or respiratory diseases.	4.47 ± 0.77	0.173	57.89	0.015	0.000	0.090
2.2.5 Facial skin moisture (e.g., sweating, exudation) affecting tape adhesion.	4.68 ± 0.48	0.102	68.42	0.012	0.000	0.090
2.2.6 Non-supine positioning(e.g., semi-recumbent, sitting, prone)	4.26 ± 0.73	0.172	42.10	0.008	0.000	0.090
2.2.7 Use of oral tracheal intubation	4.74 ± 0.45	0.096	73.68	0.048	0.001	0.090
2.2.8 Duration of tracheal intubation greater than 5 days. (34)	4.68 ± 0.48	0.102	68.42	0.021	0.001	0.090
2.2.9 GCS score ≥9 and <13 (35)	4.79 ± 0.42	0.087	78.95	0.077	0.002	0.090
2.2.10 Sedation score RASS > 1 (36)	4.74 ± 0.45	0.096	73.68	0.041	0.001	0.090
2.2.11 Muscle strength in limbs > grade 3 (37)	4.42 ± 0.51	0.115	42.10	0.100	0.003	0.090
2.2.12 CPOT score > 3 (38)	4.84 ± 0.37	0.077	84.21	0.130	0.004	0.090
2.2.13 Use of benzodiazepines (e.g., midazolam, due to the risk of delirium)	4.89 ± 0.32	0.064	89.47	0.165	0.005	0.090
2.2.14 Previous episodes of delirium during ICU stay	4.95 ± 0.23	0.046	94.47	0.222	0.006	0.090
2.2.15 Presence of ventilator weaning fatigue	4.42 ± 0.61	0.137	47.37	0.018	0.001	0.090
2.2.16 Previous history of UEE	4.68 ± 0.48	0.102	68.42	0.028	0.001	0.090
2.2.17 Position changes, such as turning	4.47 ± 0.61	0.137	52.63	0.035	0.001	0.090
2.3 Communication and Collaboration	4.74 ± 0.45	0.096	73.68	0.283	0.043	0.015
2.3.1 Ineffective communication and collaboration between doctors and nurses	4.84 ± 0.38	0.077	84.21	0.307	0.013	0.013
2.3.2 Ineffective communication and collaboration among nurses	4.79 ± 0.42	0.087	78.95	0.192	0.008	0.013
2.3.3 Patient cooperation difficulties due to agitation, delirium, or communication barriers.	4.89 ± 0.32	0.064	89.47	0.501	0.021	0.013
2.4 Work Environment	4.74 ± 0.45	0.096	73.68	0.130	0.0200	0.015
2.4.1 Inadequate lighting in the ward	4.68 ± 0.48	0.102	68.42	0.235	0.005	0.017
2.4.2 Crowded ward environment	4.63 ± 0.60	0.129	68.42	0.113	0.002	0.017
2.4.3 Excessive ambient noise in the ward	4.63 ± 0.50	0.107	68.42	0.180	0.004	0.017
2.4.4 Inappropriate ward temperature (too high or too low)	4.68 ± 0.48	0.102	68.42	0.115	0.002	0.017
2.4.5 Incomplete basic monitoring facilities at the bedside	4.79 ± 0.42	0.087	78.95	0.357	0.007	0.017
3. Inadequate Supervision	4.79 ± 0.42	0.064	89.47	0.257	0.257	0.025
3.1 Insufficient Supervision	4.89 ± 0.32	0.064	89.47	0.313	0.080	0.014
3.1.1 Deficiencies in supervision, including training, management oversight, and quality improvement	5.00	0	100.00	1.000	0.080	—
3.2 Inappropriate Work Arrangements and Plans	4.95 ± 0.23	0.046	94.74	0.492	0.126	0.014
3.2 Inappropriate Work Arrangements and Plans	4.68 ± 0.48	0.102	68.42	0.3324	0.0419	0.0127
3.2.1 High frequency of night shift assignments for clinicians	4.68 ± 0.48	0.102	68.42	0.342	0.043	0.013
3.2.2 Overtime or temporary shift changes for clinicians	4.95 ± 0.23	0.046	94.74	0.492	0.126	0.014
3.2.3 Inequitable work arrangements at different competency levels.	4.58 ± 0.61	0.133	63.16	0.326	0.041	0.013
3.3 Uncorrected Existing Problems	4.79 ± 0.42	0.087	78.95	0.196	0.050	0.014
3.3.1 Defects in the current medical workflow.	4.89 ± 0.32	0.064	89.47	0.259	0.013	0.011
3.3.2 Failure to promptly correct unsafe behaviors of clinicians	5.00	0	100.00	0.491	0.025	0.011
3.3.3 Failure to timely improve related auxiliary tools (including tube fixation devices or restraints)	4.89 ± 0.32	0.064	89.47	0.250	0.013	0.011
4. Organizational Influence	4.52 ± 0.50	0.096	73.68	0.144	0.144	0.025
4.1 Resource Management	4.74 ± 0.45	0.096	73.68	0.301	0.043	0.010
4.1.1 High proportion of newly hired clinicians	4.95 ± 0.23	0.046	94.74	0.383	0.017	0.011
4.1.2 Insufficient staffing of clinicians	4.89 ± 0.32	0.064	89.47	0.282	0.012	0.011
4.1.3 Insufficient quantity of protective restraint devices	4.74 ± 0.45	0.096	73.68	0.170	0.007	0.011
4.1.4 Quality of protective restraint devices does not meet national standards	4.68 ± 0.48	0.102	68.42	0.164	0.007	0.011
4.2 Work Systems and Procedures	4.89 ± 0.32	0.064	89.47	0.534	0.077	0.010
4.2.1 The hospital has not established relevant systems, procedures, or protocols for preventing UEE	4.95 ± 0.23	0.046	94.74	0.500	0.038	0.000
4.2.2 The hospital has not updated or revised relevant systems, procedures, and protocols for preventing UEE in line with current work conditions.	4.95 ± 0.23	0.046	94.74	0.501	0.039	0.000
4.3 Organizational Climate	4.89 ± 0.32	0.064	89.47	0.165	0.024	0.010
4.3.1 The department has not planned occupational safety meetings addressing UEE prevention	4.68 ± 0.48	0.102	68.42	0.187	0.004	0.024
4.3.2 The department lacks incentives to encourage clinicians to provide suggestions for preventing UEE	4.63 ± 0.60	0.129	68.42	0.118	0.003	0.024
4.3.3 Department management does not engage clinicians in discussions about work norms for preventing UEE	4.68 ± 0.58	0.124	73.68	0.348	0.008	0.024
4.3.4 The department does not plan operational drills to prevent UEE of tracheal intubation.	4.89 ± 0.32	0.064	89.47	0.347	0.008	0.024

CV, coefficient of variation; CR, consistency ratio.

### 3.7 Indicator weight

The consistency of the judgment matrices was assessed using the CR. The CR for the first-level indicator matrix was 0.004. For the second-level indicators, CR values ranged from 0.000 to 0.052, and for the third-level indicators, they ranged from 0.000 to 0.026. All CR values were below the acceptable threshold of 0.1, indicating that the judgment matrices demonstrated satisfactory consistency.

### 3.8 Content validity

The Level 1 indicators showed the highest content validity (S-CVI/Ave = 0.987), whereas the S-CVI/Ave values for the Level 2 indicators, Level 3 indicators, and the overall scale were 0.858, 0.821, and 0.835, respectively. The I-CVI of the Level 3 indicators ranged from 0.526 to 1.000, and 21 items had an I-CVI below 0.78, suggesting that further revision or deletion should be considered. The item-level and scale-level content validity results are presented in Table 4.

**Table 4:** Content validity of the indicator system.

Level	Number of items	I-CVI range	S-CVI/Ave
Level 1 indicators	4	0.947–1.000	0.987
Level 2 indicators	13	0.632–1.000	0.858
Level 3 indicators	63	0.526–1.000	0.821
Overall	80	0.526–1.000	0.835

## 4 Discussion

### 4.1 Methodological rigor and scientific foundation of the indicator system

The Human Factors Analysis and Classification System (HFACS), originally developed by Shappell and Wiegmann (2000) for aviation safety, provides a systematic framework for analyzing the role of human factors in adverse events. Its four-level structure enables a comprehensive and layered understanding of error causation, making it increasingly applicable to patient safety research in healthcare settings (39, 40). In the present study, HFACS served as the theoretical foundation for constructing the UEE risk assessment indicator system, complemented by a systematic literature review and a retrospective review of institutional UEE cases. This multi-source approach ensured that the initial indicator pool captured both evidence-based risk factors and context-specific clinical realities.

The scientific rigor of the indicator system was further reinforced by the methodological quality of the Delphi process. The expert panel demonstrated strong engagement, as reflected in the high questionnaire response rates (100% in Round 1% and 95% in Round 2) and the substantial proportion of experts providing qualitative feedback (70.0% and 31.6%, respectively). The expert authority coefficients (Cr = 0.893 and 0.915) exceeded the recommended threshold of 0.70, indicating that the panel possessed sufficient expertise to provide credible judgments. However, it should be noted that the Kendall's *W* values (0.242 and 0.254) indicated only a low level of consensus, rather than strong convergence. This finding suggests that although the experts generally agreed on the importance of most indicators, there was notable variation in their assessments highlighting the complexity of UEE risk factor identification and the need for future empirical validation. Overall, the I-CVI and S-CVI/Ave mostly met recommended thresholds and provided preliminary support for the content validity of the final indicator system, although 21 tertiary indicators showed suboptimal I-CVI values. While some indicators had suboptimal I-CVI values, they remained meaningful considering the HFACS framework, domestic clinical context, and experts' written feedback. For Delphi indicator screening, CV, mean importance score and full-score rate were used as primary deletion criteria, with CVI as a supplementary metric.

The Analytic Hierarchy Process (AHP) was employed to assign relative weights to the indicators. All consistency ratios (CR < 0.1) for the judgment matrices fell within the acceptable range, supporting the internal consistency of the weight assignments. Nevertheless, the preliminary nature of the system must be acknowledged; the derived weights reflect expert consensus rather than empirical evidence, and their predictive utility remains to be tested in clinical practice.

Adapting HFACS for prospective risk identification is defensible when inverted as a heuristic aligned with Reason's Swiss Cheese Model, identifying current systemic deficiencies rather than merely classifying past events. This reframing positions our tool as a screening heuristic, not a predictive algorithm. Accordingly, the high proximal weight assigned to Clinician Unsafe Behavior (0.448) reflects AHP's mathematical tendency to prioritize directly actionable precursors over distal factors, compounded by our predominantly nursing panel's clinical focus. Critically, because patient-level indicators (e.g., age, sedation score) and organizational indicators (e.g., staffing, policy completeness) operate on distinct analytical units, we disaggregated them into two components: bedside risk screening vs. systemic safety audit. This resolves dimensional confounding and explains why patient-level global weights are mathematically attenuated through hierarchical multiplication (e.g., “age > 65 years” at 0.0006), rendering cross-component comparisons invalid. This two-component framework offers a comprehensive structure but remains a consensus-based heuristic requiring prospective validation to establish predictive utility and clinical feasibility.

The institutional UEE incidence rate of 2.89% observed in our retrospective case review falls below the pooled prevalence of 6.69% (95% CI: 5.29–8.24) reported in a recent meta-analysis (3). This discrepancy may reflect differences in case mix, sedation practices, nurse-to-patient ratios, and institutional reporting thresholds, underscoring the importance of context-specific risk assessment and the need for caution when applying pooled epidemiological estimates to individual clinical settings.

### 4.2 Content and weight analysis of the risk assessment system for UEE in ICU patients

#### 4.2.1 Unsafe acts of clinicians

Our findings are consistent with a recent Delphi-based study that developed a 43-item evidence-based nursing program for UEE prevention, which similarly emphasized the multidimensional nature of UEE risk factors encompassing sedation, analgesia, delirium, and protective restraints (41).

Unsafe acts of clinicians comprises two main categories: errors and violations. Our findings indicated that unsafe acts of clinicians carried the highest weight among the first-level indicators, with violation receiving the highest weight among the three second-level indicators; skill errors and decision-making errors were assigned equal weight values.

Regarding violations, clinicians are central to procedural execution, and their actions are critical contributors to the occurrence of unsafe events. Examples identified in this study included clinicians performing high-risk procedures alone (e.g., replacing bite blocks or fixation tape), failing to promptly assess tracheal intubation status, conducting inadequate airway assessment and handover, and applying restraints ineffectively. Crucially, HFACS identifies unsafe acts to uncover systemic vulnerabilities, not to assign individual blame. Per Reason's Swiss Cheese Model, errors originate from latent conditions in workflow, training, and organizational culture. Accordingly, this dimension's high weight signals system-level interventions—examining staffing, workload, training adequacy, equipment usability, and decision support- rather than disciplinary action. “Performing high-risk procedures alone” may reflect understaffing or ambiguous protocols, not negligence; “failure to assess extubation criteria” may indicate absent standardized tools or poor communication. By embedding individual behaviors within broader contextual factors across preconditions, supervision, and organizational levels, HFACS guides safety investigations toward actionable systemic improvements. While restraint use is often intended to prevent tube dislodgement, evidence suggests that judicious reduction of physical restraints may decrease the occurrence of adverse events in intubated patients (42), underscoring the importance of continuous risk assessment and timely decision-making regarding the need for and timing of restraint application. A meta-analysis demonstrated that physical restraint is associated with increased UEE risk [OR = 3.44, 95% CI (2.52–4.72)] (43), challenging the assumption that restraint uniformly prevents UEE. Conversely, restraint reduction programs have been shown to effectively decrease UEE incidence [OR = 2.42, 95% CI (1.65–3.56)] (44). Furthermore, physical restraint is a significant modifiable risk factor for delirium [OR = 15.46, 95% CI (6.66–20.01)] (45) and may paradoxically exacerbate agitation (46). Accordingly, our inclusion of restraint indicators does not endorse routine restraint, but prompts evaluation of necessity, prioritizing evidence-based alternatives: (1) light sedation with daily interruption; (2) delirium screening and multimodal prevention; (3) communication tools; (4) early mobilization; (5) family engagement; and (6) standardized criteria with mandatory daily reassessment. When indicated, restraints should be least restrictive, shortest duration, with ongoing risk-benefit evaluation, aligning with current clinical practice guidelines (47). A systematic review of UEE in adult ICUs identified that suboptimal airway security practices—including inadequate tube fixation, failure to monitor cuff pressure, and absence of standardized extubation readiness assessments-are consistently reported as contributing factors to UEE events (48). High-quality, standardized airway management is fundamental to UEE prevention. This includes routine assessments of tracheal intubation depth, cuff pressure monitoring, fixation effectiveness evaluation, and adherence to standardized protocols.

Skill errors primarily stem from inadequate clinical competencies or improper execution of procedures by clinicians, such as limited clinical experience in managing intubated patients, delayed recognition of equipment alarms, and inadequate patient education and family education, all of which may increase UEE risk.

Decision-making errors encompass errors in clinical reasoning and judgment, including inappropriate selection of interventions, misjudgment of extubation readiness, and failure to adequately assess procedural risks. Specific examples from our system include incorrect choice of endotracheal tube size, inappropriate sedation and analgesia strategies, and premature or delayed decisions regarding extubation. A meta-analysis by Li et al. (3) demonstrated that approximately 50% of patients who experience UEE tolerate tube removal without requiring reintubation, suggesting that planned extubation may be unnecessarily delayed in a substantial proportion of critically ill patients. This highlights the clinical importance of accurate and timely assessment of extubation criteria to prevent both UEE and prolonged unnecessary intubation.

#### 4.2.2 Preconditions for unsafe acts

Preconditions for unsafe acts refer to the conditions that directly precipitate unsafe actions, encompassing personnel-related, environmental, and technological factors. Among the four second-level indicators within this domain, the weights ranked in descending order were as follows: clinician condition, communication and collaboration, patient condition, and working environment.

Regarding personnel-related factors, the indicator system encompasses clinician condition, patient condition, and communication and collaboration among healthcare providers. Numerous studies have documented that suboptimal physiological and psychological states of clinicians can impair their performance and contribute to UEE events (49). The ICU environment presents unique challenges, and the communication barriers imposed by tracheal intubation further complicate patient care. Accordingly, strategies such as providing psychological support to patients, enhancing comfort, and facilitating alternative communication methods are key to reducing UEE (41).

Patient-specific factors also play a significant role in predisposing to unsafe acts that may result in UEE. These include age, gender, comorbidities, medication use, severity of illness, and type of tracheal intubation. Male patients, in particular, have been identified as a high-risk group for UEE (34). A recent survey of Society of Critical Care Medicine members identified delirium as the highest-ranked UEE risk factor in adult ICUs (48).

Effective teamwork and interprofessional collaboration are fundamental to safe clinical practice. Studies suggest that peer support and collegial cooperation significantly promote safe behavior among healthcare staff (50). In particular, effective communication and collaboration between the responsible nurse and attending physician are critical to preventing delayed extubation (15).

The work environment is an important external factor influencing UEE in ICU patients. With regard to lighting and monitoring facilities, adequate lighting enables healthcare providers to accurately observe patient status and tube fixation, while comprehensive bedside monitoring equipment facilitates timely detection of abnormalities and helps prevent UEE. Inappropriate temperature and humidity can compromise patient comfort, potentially increase agitation, and thus indirectly elevate the risk of UEE.

#### 4.2.3 Inadequate supervision

Inadequate supervision represents a management-level contributor to UEE. Among its three second-level indicators, the weights ranked in descending order were: inappropriate work arrangements and plans, insufficient supervision, and failure to address existing problems.

Inappropriate work arrangements and plans carried the highest weight within this dimension. Specifically, a high frequency of night shifts, overtime, and temporary shift changes can lead to physical and mental fatigue among clinicians, thereby increasing the risk of UEE (34). Yeh et al. (51) found that insufficient clinical experience among ICU nurses can contribute to a higher incidence of UEE. These findings underscore the importance of providing targeted, competency-based training for clinical staff at different professional levels, alongside making reasonable work assignments that account for varying levels of clinical experience.

Insufficient supervision encompasses failures in training, guidance, and monitoring of clinicians' practice. When training is absent, inadequate, or lacking in diversity and intensity, it fails to equip staff with the necessary knowledge to recognize and mitigate unsafe events, and does little to enhance their vigilance. Conversely, adequate supervision is essential to maintaining high standards of care and preventing UEE.

With respect to uncorrected existing problems, systemic weaknesses in hospital workflows can create latent conditions that predispose to adverse events. If clinicians' unsafe actions are not promptly identified and corrected through an effective feedback and remediation system, these behaviors may persist and continue to jeopardize patient safety. Furthermore, if tracheal intubation-related tools are not updated in line with advances in medical technology and evolving clinical needs, they may indirectly increase the risk of UEE. However, equipment improvements should accompany broader commitments to minimizing restraint use through better sedation, delirium prevention, and patient communication, not as a standalone fix.

Collectively, these findings highlight that clinical managers bear a critical responsibility for nursing quality and patient safety. Strengthening the nursing management system through continuous quality improvement, proactive risk identification, and timely corrective actions is essential to reducing the incidence of UEE and safeguarding patient rights.

#### 4.2.4 Organizational influence

Organizational influence refers to the impact of hospital-level management decisions on the occurrence of UEE. Among the three second-level indicators in this domain, work systems and procedures carried the highest weight, followed by resource management and organizational climate, which were assigned equal weights.

Well-established work systems and procedures are fundamental to guiding clinicians in performing medical tasks correctly and consistently. When a hospital has not developed or updated its systems, protocols, and guidelines for UEE prevention, or when these documents fail to reflect current clinical realities, they lose their effectiveness in regulating clinical behaviors and mitigating risks. Regular review and revision of such protocols are therefore essential to maintaining their relevance and utility.

Regarding resource management, insufficient staffing levels, and inadequate resources to UEE prevention are closely associated with increased UEE risk. Ensuring appropriate nurse-to-patient ratios and providing necessary material resources are critical organizational responsibilities that directly influence patient safety.

Organizational climate exerts a subtle but pervasive influence on the attitudes and behaviors of clinical staff. A positive organizational climate-characterized by open communication, shared safety values, and visible leadership commitment-can enhance awareness of workplace hazards, foster vigilance toward unsafe events, and improve compliance with evidence-based safety practices, collectively contributing to a reduction in UEE risk.

## 5 Limitations

Several limitations of this study warrant consideration. Expert consensus was low (Kendall's *W* = 0.242–0.254) and did not meaningfully improve between rounds, suggesting the indicator weights should be viewed as provisional and exploratory. The system has not undergone any psychometric evaluation, and its reliability and validity remain unestablished. Although several indicators pertain to physician-level decisions, the panel comprised predominantly nursing professionals, with no intensivists, respiratory therapists, human-factors specialists, or patients—a composition that may have introduced disciplinary bias. Both the panel and case review were drawn from a single institution in China, limiting generalizability.

The system mixes patient-, unit-, and organizational-level indicators within a single framework, complicating interpretation. HFACS was originally designed for retrospective analysis, and its prospective use lacks empirical validation. The 63 indicators may prove impractical for routine bedside use, and no feasibility testing has been conducted. Several indicators rely on sensitive clinician information, posing practical and ethical challenges. The literature search concluded in January 2023, excluding recent evidence. Finally, the narrow importance score range (4.47–5.00) suggests a ceiling effect, potentially reflecting social desirability or acquiescence bias that limits the discriminative capacity of the ratings.

In summary, this remains a preliminary consensus-based framework requiring substantial refinement and prospective validation before clinical translation.

## 6 Conclusion

This study developed a preliminary, consensus-based risk assessment indicator system for UEE in adult ICU patients, grounded in the HFACS framework and refined through a literature review, retrospective case analysis, two rounds of Delphi expert consultation, and the Analytic Hierarchy Process. The system offers a structured, theory-driven framework for understanding and organizing UEE-related risk factors across multiple levels. However, it is important to emphasize that this is a consensus-based framework for safety audits, training needs assessment, and root cause analysis, rather than a validated clinical tool. Future research should focus on refining the indicator set, conducting prospective validation studies to evaluate its reliability and predictive validity, and simplifying the system for practical bedside use. Such efforts will help determine its clinical utility in supporting UEE prevention and improving patient safety.

## Data Availability

The raw data supporting the conclusions of this article will be made available by the authors, without undue reservation.
