# Supplementary material for: Development of an HFACS-based risk assessment indicator system for unplanned endotracheal extubation in adult ICU patients: a Delphi and AHP study
Source: Front Med (Lausanne). 2026 Oct 28;13:1922086. doi: 10.3389/fmed.2026.1922086 (PMC13751363; doi:10.3389/fmed.2026.1922086)
Supplement: Supplementary file 1 [file Supplementaryfile1.docx]

**Supplementary Material**

### Supplementary Table S1：

**Databases and Search Strategies**

| **Databases** | **Search Strategies** |
| --- | --- |
| **PubMed** | ("intensive care units"[MeSH] OR "critical care"[MeSH] OR "intensive care" OR ICU) AND ("intubation, intratracheal"[MeSH] OR "endotracheal intubation" OR "mechanical ventilation") AND ("unplanned extubation" OR "self-extubation" OR "accidental extubation" OR "device removal" OR "dislodgement") |
| **EMBASE** | ('intensive care'/exp OR 'critical care'/exp OR ICU) AND ('endotracheal intubation'/exp OR 'mechanical ventilation'/exp) AND ('unplanned extubation'/exp OR 'self extubation' OR 'accidental extubation') |
| **Web of Science** | TS=((ICU OR "intensive care" OR "critically ill")) AND TS=("endotracheal intubation" OR "mechanical ventilation") AND TS=("unplanned extubation" OR UEE OR "self-extubation" OR "accidental extubation") |
| **CINAHL** | (MH "Intensive Care Units" OR MH "Critical Care" OR ICU) AND (MH "Intubation, Intratracheal" OR "endotracheal intubation") AND ("unplanned extubation" OR "self-extubation") |
| **CNKI** | (SU=重症监护 OR SU=ICU OR SU=危重症) AND (SU=气管插管 OR SU=机械通气) AND (SU=非计划性拔管 OR SU=意外拔管 OR SU=自行拔管) |
| **Wanfang** | 主题:(重症监护 OR ICU OR 危重症) AND 主题:(气管插管 OR 机械通气) AND 主题:(非计划性拔管 OR 意外拔管) |
| **VIP** | (T=重症监护 OR T=ICU) AND (T=气管插管) AND (T=非计划性拔管) |

### Supplementary Table S2：

### Literature screening process and reasons for exclusion

PubMed(n = 118）

Embase(n＝159）

Web of Science(n = 175)

CINAHL(n = 50)

CNKI(n＝621）

Wanfang(n＝1196）

VIP(n＝229)

Records identified from databases searching

（n＝2548）

**Identification**

**Screening**

Records after duplicathes remove(n =1810)

Reports excluded(n = 1748)

Full-text articles excluded(n =43)

1.Ineligible study population(n＝25）

2.Inconsistent research concepts(n＝8）

3.Mismatched clinical scenarios(n＝10）

Full-text articles assessed for eligibility(n =62)

**Included**

Studies included

(n =19 )

Fig. 1 Literature screening process and reasons for exclusion

### **General Characteristics of the Included Literature**

| No. | authors | year | Title | Type of literature | Quality Rating |
| --- | --- | --- | --- | --- | --- |
| 1 | Higgs et al. | 2017 | Guidelines for the management of tracheal intubation in critically ill adults | guideline | High |
| 2 | Devlin et al. | 2018 | Clinical Practice Guidelines for the Prevention and Management of Pain, Agitation/Sedation, Delirium, Immobility, and Sleep Disruption in Adult Patients in the ICU | guideline | High |
| 3 | Murray et al. | 2016 | Clinical Practice Guidelines for Sustained Neuromuscular Blockade in the Adult Critically Ill Patient | guideline | High |
| 4 | Society Of Critical Care Medicine Chinese Medical Association | 2018 | Guidelines for analgesia and sedation in Chinese adult ICUs | guideline | Moderate |
| 5 | Wang, Y et al. | 2019 | consensus on preventing unplanned extubation care for adult transoral endotracheal intubation | consensus | Moderate |
| 6 | Garrett | 2016 | Best Practices for Managing Pain, Sedation, and Delirium in the Mechanically Ventilated Patient | best practice | High |
| 7 | Liu, Y. F. et al. | 2020 | Summary of evidence-based prevention and management of unplanned extubation in adult ICU patients | summary of evidence | High |
| 8 | Da Silva et al. | 2012 | Systematic review and evidence-based recommendations for unplanned extubation of tracheal intubation in ICUs | systematic review | Moderate |
| 9 | Kiekkas | 2013 | Clinical review of unplanned extubation in critically ill adults | systematic review | N/A (narrative review) |
| 10 | Gardner | 2005 | Best practice in stabilisation of oral endotracheal tubes | systematic review |  |
| 11 | Minhas et al. | 2015 | Effect of protocolized sedation on clinical outcomes in mechanically ventilated intensive care unit patients | systematic review | Low |
| 12 | Chen et al. | 2014 | Effectiveness of daily interruption of sedation in sedated patients with mechanical ventilation in ICU | systematic review | Moderate |
| 13 | King et al. | 2012 | Safety and monitoring of unplanned extubation in mechanically ventilated patients | systematic review | Low |
| 14 | Aitken et al. | 2018 | Protocol-directed sedation versus non-protocol-directed sedation in mechanically ventilated intensive care adults and children | systematic review | High |
| 15 | Wang FY | 2022 | Risk factors for unplanned extubation in ICU patients with endotracheal intubation: a meta-analysis | systematic review | High |
| 16 | Wu JH | 2021 | Risk assessment tools for unplanned extubation of endotracheal intubation: a scoping review | systematic review | N/A (background reference) |
| 17 | Lu ZH | 2022 | Development of a risk assessment scale for unplanned endotracheal extubation in adult patient | scale development | High |
| 18 | Zhang P | 2021 | Construction of a risk assessment scale for unplanned extubation in patients with endotracheal intubation | scale development | High |
| 19 | Fisher DF | 2014 | Comparison of commercial and noncommercial endotracheal tube-securing devices | randomized controlled study | High |

1. Higgs A, McGrath BA, Goddard C, et al. Guidelines for the management of tracheal intubation in critically ill adults. Br J Anaesth. 2018;120(2):323-352.
2. Devlin JW, Skrobik Y, Gélinas C, et al. Clinical practice guidelines for the prevention and management of pain, agitation/sedation, delirium, immobility, and sleep disruption in adult patients in the ICU. Crit Care Med. 2018;46(9):e825-e873.
3. Murray MJ, DeBlock H, Erstad B, et al. Clinical practice guidelines for sustained neuromuscular blockade in the adult critically ill patient. Crit Care Med. 2016;44(11):2079-2103.
4. 中华医学会重症医学分会. 中国成人ICU镇痛和镇静治疗指南. 中华重症医学电子杂志. 2018;4(2):57-83.
5. 天津市护理质控中心. 预防成人经口气管插管非计划性拔管护理专家共识. 中华护理杂志. 2019;54(6):822-828.
6. Garrett JH. Best practices for managing pain, sedation, and delirium in the mechanically ventilated patient. Crit Care Nurs Clin North Am. 2016;28(2):143-155.
7. 刘云访, 喻姣花, 李素云, 等. 成人ICU患者非计划性拔管预防与管理的最佳证据总结. 中华护理杂志. 2020;55(10):1590-1596.
8. Da Silva PSL, Fonseca MCM. Unplanned endotracheal extubations in the intensive care unit: systematic review, critical appraisal, and evidence-based recommendations. Anesth Analg. 2012;114(5):1003-1014.
9. Kiekkas P, Aretha D, Panteli E, et al. Unplanned extubation in critically ill adults: clinical review. Nurs Crit Care. 2013;18(3):123-134.
10. Gardner A. Best practice in stabilisation of oral endotracheal tubes. Aust Crit Care. 2005;18(3):111-118.
11. Minhas MA, Velasquez AB, Kaul V, et al. Effect of protocolized sedation on clinical outcomes in mechanically ventilated intensive care unit patients: a systematic review and meta-analysis. Crit Care Med. 2015;43(12):2457-2466.
12. Chen Y, Wang L, Zhang Q. Effectiveness of daily interruption of sedation in sedated patients with mechanical ventilation in ICU: a systematic review. Chin J Nurs. 2014;49(9):1086-1091.
13. King G, Elliott D. Safety and monitoring of unplanned extubation in mechanically ventilated patients. J Clin Nurs. 2012;21(15-16):2235-2244.
14. Aitken LM, Bucknall T, Kent B, et al. Protocol-directed sedation versus non-protocol-directed sedation in mechanically ventilated intensive care adults and children. Cochrane Database Syst Rev. 2018;(4):CD009771.
15. 王法颍,杨子,彭泽宇,等. ICU气管插管患者非计划拔管危险因素的Meta分析[J]. 中国卫生质量管理,2022,29(4):62-67. DOI:10.13912/j.cnki.chqm.2022.29.04.17.
16. 伍金花,黄海星,刘智利,等. 气管插管非计划拔管风险评估工具的范围综述[J]. 中华护理杂志,2021,56(11):1752-1757. DOI:10.3761/j.issn.0254-1769.2021.11.026.
17. 鲁志卉,王颖,黄子菁,等. 成人气管插管非计划性拔管风险评估量表的构建[J]. 中国护理管理,2022,22(6):893-898. DOI:10.3969/j.issn.1672-1756.2022.06.018.
18. 张萍,刘丽萍. 气管插管患者非计划拔管风险评估量表的构建[J]. 护理学报,2021,28(21):18-22. DOI:10.16460/j.issn1008-9969.2021.21.018.
19. Fisher DF, Chenelle CT, Marchese AD, et al. Comparison of commercial and noncommercial endotracheal tube-securing devices. Respir Care. 2014;59(4):531-537.
