# Supplementary material for: Development of an HFACS-based risk assessment indicator system for unplanned endotracheal extubation in adult ICU patients: a Delphi and AHP study
Source: Front Med (Lausanne). 2026 Oct 28;13:1922086. doi: 10.3389/fmed.2026.1922086 (PMC13751363; doi:10.3389/fmed.2026.1922086)
Supplement: Supplementary file 2 [file Supplementaryfile2.docx]

**Supplementary Material**

Flowchart of the Delphi Study Process

PREPARATORY PHASE

Literature Search (n=2,548 records)

Screening & Eligibility Assessment

19 Studies Included (four guidelines, one expert consensus，one best practice, one summary of evidence ,nine systematic review ,two scale development ,one randomized controlled study)

Retrospective Case Review

(January 2018 -December 2022)

17 UEE Events Identified (Institutional incidence rate: 2.89%)

Indicator Extraction & Integration (Mapped onto HFACS 4-level framework)

Initial Indicator Pool:

4 Primary ,13 Secondary ,83 Tertiary Indicators

ROUND2 DELPHI CONSULTATION

ROUND 1 DELPHI CONSULTATION

Expert Panel: n=19 (1 lost to follow-up)

Questionnaires Distributed: 20，

Returned: 19，Response Rate: 95%

Experts Providing Written Suggestions:

6 (31.6%)

Experts Statistical Analysis: Mean ≥ 4.0, Full Score Rate > 20%, CV ≤ 0.25

Experts Revisions Based on Expert Feedback:

6 indicators deleted

3 indicators added

5 indicators merged	3 indicators revised

Final Indicator Pool:

4 Primary,13 Secondary, 63 Tertiary Indicators

Expert Panel: n=20

Questionnaires Distributed: 20，

Returned: 20，Response Rate: 100%

Experts Providing Written Suggestions:

14 (70.0%)

Experts Statistical Analysis: Mean ≥ 4.0, Full Score Rate > 20%, CV ≤ 0.25

Experts Revisions Based on Expert Feedback: 15 indicators deleted	4 indicators added	2 indicators merged	11 indicators revised

Revised Indicator Pool:

4 Primary,13 Secondary,71 Tertiary Indicators

AHP PHASE

19 Experts Completed Pairwise Comparison Matrices (Saaty's 1-9 Scale)

Individual Judgments Aggregated by Geometric Mean Method

Consistency Check: CR < 0.1 for All Matrices (Accepted)

Local & Global Weights Calculated Layer by Layer

Final Weighted Indicator System: 4 Primary,13 Secondary,63 Tertiary

Indicators with AHP-Derived Weights
