# Supplementary material for: Development of an HFACS-based risk assessment indicator system for unplanned endotracheal extubation in adult ICU patients: a Delphi and AHP study
Source: Front Med (Lausanne). 2026 Oct 28;13:1922086. doi: 10.3389/fmed.2026.1922086 (PMC13751363; doi:10.3389/fmed.2026.1922086)
Supplement: Supplementary file 3 [file Supplementaryfile3.docx]

## CREDES Checklist

### Part 1: Conducting a Delphi Study

| **Item** | **Description** | **Page in Original** |
| --- | --- | --- |
| **Rationale for the Choice of the Delphi Technique** | Justification for the choice of the Delphi technique as a method of systematically collating expert consultation and building consensus. | Introduction, p. 2-3 |
| **Planning and Design** | Planning and process adjustments, justification for modifications, and definition of consensus criteria. | Methods 2.3, p. 10-13 |
| **Planning and Process** | Adjust the Delphi technique to fit research aims and justify any modifications. | Methods 2.3.3, p. 7 |
| **Definition of Consensus** | Define a priori criteria for consensus, including guidelines for proceeding with items, thresholds for terminating the process, and procedures for handling non-consensus. | Methods 2.3.3, p. 7 |
| **Study Conduct** | Informational input review, prevention of bias, interpretation and processing of results, and external validation. | Methods 2.3, p. 10-13 |
| **Informational Input** | Review and pilot all materials provided to the expert panel to prevent bias. | Methods 2.3, p. 10-13 |
| **Prevention of Bias** | Take measures to avoid influencing experts' judgments and consider using an independent researcher if conflicts of interest exist. | Methods 2.3.2, p. 6 |
| **Interpretation and Processing of Results** | Recognize that consensus does not imply the 'correct' answer and consider non-consensus and stable disagreement as informative. | Discussion 4.1, p. 13-14 |
| **External Validation** | Have the final draft reviewed and approved by an external board or authority. | Not applicable |

### **Part 2: Reporting a Delphi Study**

| **Item** | **Description** | **Page in Original** |
| --- | --- | --- |
| **Reporting** | Purpose and rationale, expert panel criteria, description of methods, procedure flow chart, definition and attainment of consensus, results reporting, discussion of limitations, adequacy of conclusions, and publication and dissemination plan. | Supplementary Material |
| **Purpose and Rationale** | Clearly define the study's purpose and justify the use of the Delphi technique. | Abstract / Introduction, p. 2-3 |
| **Expert Panel** | Provide criteria for expert selection, recruitment details, and sociodemographic information. | Methods 2.3.2, p. 6; Table 1, p. 8 |
| **Description of Methods** | Include information on preparatory steps, survey design, data analysis, and methodological decisions. | Methods 2.2, 2.3.1, 2.3.3, 2.4, p. 5-8 |
| **Procedure** | Use a flow chart to illustrate the stages of the Delphi process, including a preparatory phase, the actual "Delphi rounds", interim steps of data processing and analysis, and concluding steps. | Supplementary Material |
| **Definition and Attainment of Consensus** | It needs to be comprehensible to the reader how consensus was achieved throughout the process, including strategies to deal with non-consensus. | Methods 2.3.3, p. 7; Results 3.5, p. 8-10 |
| **Results** | Reporting of results for each round separately is highly advisable in order to make the evolving of consensus over the rounds transparent. This includes figures showing the average group response, changes between rounds, as well as any modifications of the survey instrument such as deletion, addition or modification of survey items based on previous rounds. | Results 3.2, 3.3, 3.4, 3.5, p. 8-10; Table 2, p. 9 |
| **Discussion of Limitations** | Reporting should include a critical reflection of potential limitations and their impact on the resulting guidance. | Limitations, p. 14-15 |
| **Adequacy of Conclusions** | The conclusions should adequately reflect the outcomes of the Delphi study with a view to the scope and applicability of the resulting practice guidance. | Conclusion, p. 15 |
| **Publication and Dissemination** | The resulting guidance on good practice should be clearly identifiable from the publication, including recommendations for transfer into practice and implementation. If the publication does not allow for a detailed presentation, reference to a more detailed presentation elsewhere should be made. | Conclusion, p. 15; Supplementary material, p. 16 |
